# Simultaneous molecular detection of *Anaplasma marginale* and *Theileria annulata* in cattle blood samples collected from Pakistan-Afghanistan boarder region

**DOI:** 10.1371/journal.pone.0288050

**Published:** 2023-07-20

**Authors:** Sania Jamil, Chien-Chun Chiou, Hira Muqaddas, Hayat Ullah, Muhammad Asif, Sana Rao, Hafsa Hussain, Qandeel Fatima, Nasreen Nasreen, Sadaf Niaz, Karla Dzul-Rosado, Adil Khan, Furhan Iqbal, Chien-Chin Chen

**Affiliations:** 1 Institute of Zoology, Bahauddin Zakariya University Multan, Multan, Pakistan; 2 Department of Dermatology, Ditmanson Medical Foundation Chia-Yi Christian Hospital, Chiayi, Taiwan; 3 Department of Zoology, The Women University Multan, Multan, Pakistan; 4 Department of Zoology, Abdul Wali Khan University Mardan, Mardan, Pakistan; 5 Center for Regional Studies Hideyo Noguchi, Autonomous University of Yucatan, Merida, Yucatan, Mexico; 6 Department of Botany and Zoology, Bacha Khan University, Charsadda, Khyber Pakhtunkhwa, Pakistan; 7 Department of Pathology, Ditmanson Medical Foundation Chia-Yi Christian Hospital, Chiayi, Taiwan; 8 Department of Cosmetic Science, Chia Nan University of Pharmacy and Science, Tainan, Taiwan; 9 Ph.D. Program in Translational Medicine, Rong Hsing Research Center for Translational Medicine, National Chung Hsing University, Taichung, Taiwan; 10 Department of Biotechnology and Bioindustry Sciences, College of Bioscience and Biotechnology, National Cheng Kung University, Tainan, Taiwan; King Abdulaziz City for Science and Technology (KACST), SAUDI ARABIA

## Abstract

*Theileria annulata* (*T*. *annulata*) and *Anaplasma marginale* (*A*. *marginale*) are among the most extensively reported tick borne pathogens and are associated with huge economic losses worldwide. A total of 298 cattle blood samples were screened to report the presence of these two pathogens. The samples were collected from apparently healthy cattle (Achai, n = 155, Jersy, n = 88 and crossbred, n = 55) in Bajaur district of Khyber Pakhtunkhwa (KPK) during June and July of 2022. A total of 31 out of 298 cattle (10.4%) were found infected with *T*. *annulata* as PCR amplified a 156 base pair fragment from *Tams-1* gene of *T*. *annulata* from their blood. While 16/298 animals (5.4%) were found infected with *A*. *marginale* as they amplified a 382 base pair fragment specific for *msp5* gene of this bacterium. Three animals (1%) were found co infected. Cattle susceptibility to *T*. *annulata* infection was significantly higher than *A*. *marginale* infection (P < 0.001). Phylogenetic analysis revealed that Pakistani isolates of both detected pathogen clustered together and were closely related isolates from worldwide countries. Prevalence of *T*. *annulata* varied significantly among the sampling sites (P = 0.05) while no such association was observed for *A*. *marginale* among the tested cattle. Epidemiological data analysis revealed that none of the studied risk factors was found associated either with the prevalence of *T*. *annulata* or *A*. *marginale* (P > 0.05) among enrolled cattle. In conclusion, our study has revealed a relatively higher prevalence of *T*. *annulata* than *A*. *marginale* in cattle from the Bajaur district in KPK. This information is important for improving the productivity of the livestock sector, which is one of the main sources of income in the country. It is recommended that this data be taken into account for the development and implementation of effective tick control programs, as well as for the improvement of livestock management practices to prevent and manage TBDs in Pakistan.

## Introduction

Pakistan is an agricultural country where annual economic growth is largely dependent upon livestock. Majority of the population live a rural life and each family keep livestock and poultry for their subsistence and meeting other requirements of life [1]. During the financial year 2018–19, 18.5% of the national gross domestic product (GDP) was contributed by agriculture in which the share of livestock was 11.2% [2]. Production of healthy and robust animals with improved animal welfare and preserving genetic diversity are the aims of modern livestock production systems in Pakistan [3]. Poor management, lack of nutrition, low inputs, inadequate artificial insemination service and diseases are the main reasons of low productivity of cattle in Pakistan [4]. Ticks are among the most commonly reported vectors from Pakistan and are involve, ticks populations have been reported in different frequencies ranging from 6.99% to 80% in large in the transmission of a number of pathogens to cattle including *Theileria annulata* and *Anaplasma marginale* [5].

Tropical theileriosis is a lymphoproliferative disease that is caused by the intracellular schizonts of *T*. *annulata*, a Protozoan parasite from Apicomplexa. *T*. *annulata* infection in ruminants onsets with the transmission of sporozoites stage of the parasite by the ticks during the blood meal [6]. These sporozoites preferentially invade the B cells and monocytes of the cattle and causes severe morbidity and mortality in livestock, resulting in reduced meat and milk production that lead to significant economic losses [7]. *T*. *annulata* infected bovine cells share several hallmarks of cancer such as deregulation of energetic metabolism, resistance to apoptosis, uncontrolled proliferation and acquiring an invasive phenotype. The chemotherapy for this disease is done by the application of buparvaquone [8].

*Anaplasma marginale*, a member of the family Anaplasmataceae, is an obligate tick borne intracellular Gram-negative alpha proteobacterium that targets the erythrocytes of the host that includes a number of animals including cattle [9]. *A*. *marginale* is responsible for high mortality rates in cattle herds in tropical, subtropical and temperate regions of the world [10]. Infection due to *A*. *marginale* results in reduced body weight of infected animals and can cause abortion. Reduce milk and meat production is also among common symptoms post bacterial infection [4]. It has been reported that ticks including *Rhipicephalus microplus* (*R*. *microplus*), *R*. *turanicus* and *R*. *haemaphysaloides* are involve in the transmission of this bacterium to livestock [11]. While mechanical transmission of *A*. *marginale* by biting flies, blood contaminated needles and farm equipment has also been reported [5].

Although the prevalence of *T*. *annulata* and *A*. *marginale* and the associated risk factors has been reported from various geo-climatic regions of Pakistan but these tick borne pathogens has been poorly investigated in the north-western part of the Pakistan especially in Bajaur district that is bordering with Afghanistan where routine cross-border traffic is the main source of spreading the infection among livestock. The lack of data concerning the prevalence of *T*. *annulata* and *A*. *marginale* from this study area results in poor control measures. The present study identified the presence of *T*. *annulata* and *A*. *marginale* in the bovine population of Bajaur district in KPK. Furthermore, the study conducted a molecular phylogeny analysis of these pathogens and reported their epidemiological profile.

## Materials and methods

### Study area and blood sampling

Bajaur is a district in Khyber Pakhtunkhwa that lies at the Pak- Afghan border. This district is comprised of mountains and hilly areas with livestock are the major income source of the residents. Afghanistan lies in the West of Bajur while Mohmand district is in its South, lower Dir in the North and Pankora River is in the east of Bajur. The climate of this region is semi-arid and temperature fluctuates between 5–10°C during winter and summer temperatures ranges between 23 to 26°C [12].

**Fig 1:**
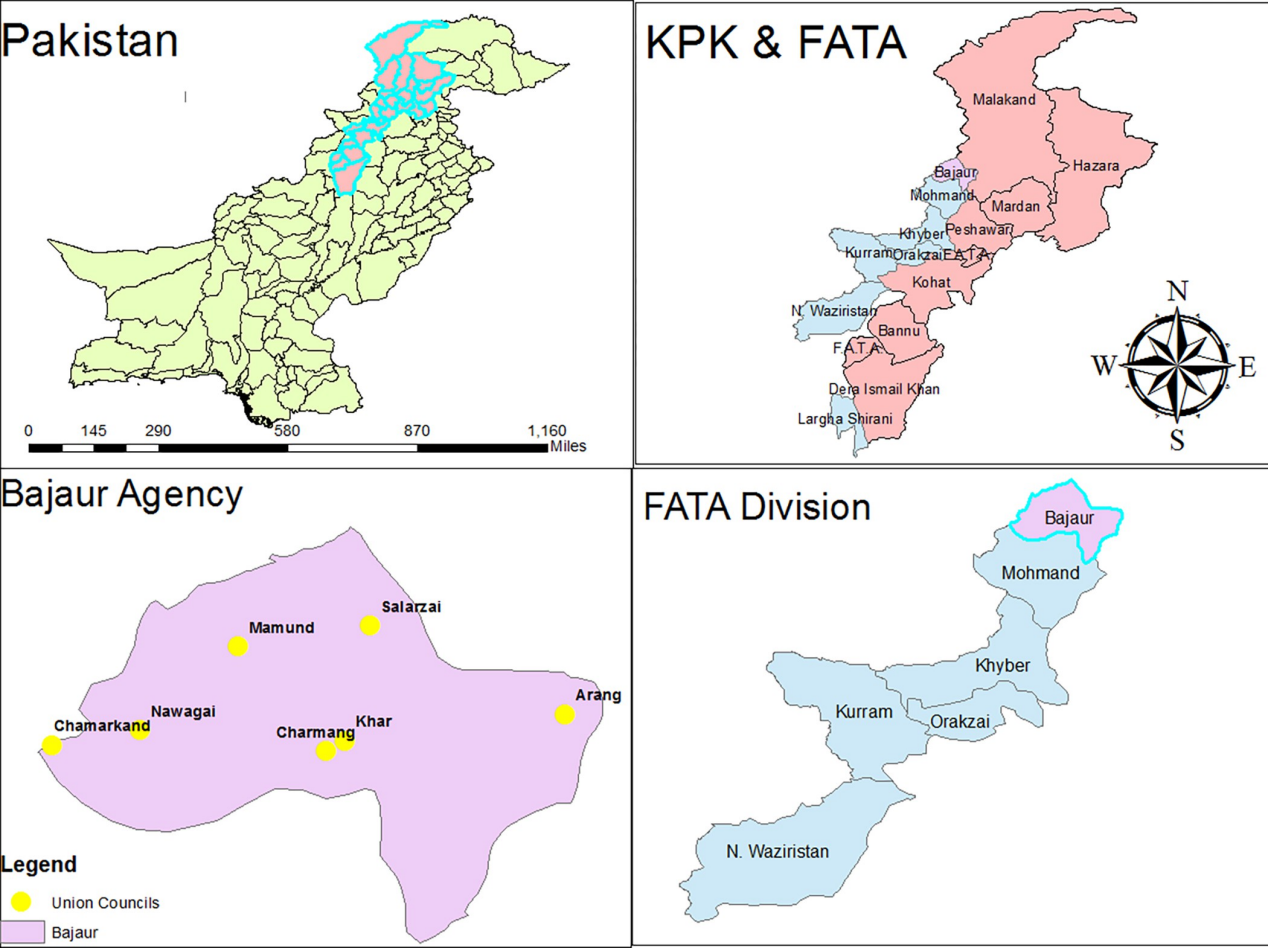
Map of Pakistan with Khyber Pakhtunkhwa province having boarders with Afghanistan at district Bajaur is highlighted in light pink color. While sampling sites are highlighted with yellow circles in magnified map of Distict Bajaur. ArcGIS 10.7.1 version was used for mapping. Shape files were downloaded from the link (https://www.diva-gis.org/gdata) and maps were saved in tiff format.

Informed consent was obtained from the livestock owners to collect the blood and epidemiological data of the animals at their farms. A total of 298 blood samples of apparently healthy cattle from three breed (Achai, n = 155, Jersy, n = 88 and crossbred, n = 55) were collected from randomly selected herds located in different parts of district Bajaur in Khyber Pakhtunkhwa (KPK) during June and July of 2022. The cattle were enrolled from Khar, Nawagai, Salaezi, Utman Khel, Chamarkand, Mamund, Arang and Charmang (Fig 1).

A predesigned questionnaire was used to collect epidemiological data (sex, age, grazing, pre-Acaricide spray, herd size, and tick burden on cattle) in order to investigate their association with the prevalence of *Theileria annulata* and *Anaplasma marginale* in cattle enrolled during present.

### DNA extraction

A volume of 3–5 ml of blood was taken from the jugular vein of each animal into an EDTA collection tube for DNA extraction. Genomic DNA was extracted from collected blood samples as described by Fatima et al. [13]. The quality of the extracted DNA was assessed by measuring the optical density at 260/280 nm (O. R. I. Reinbeker, Hamburg) and by using submerged 1% gel electrophoresis.

### PCR amplification

A multiplex PCR was performed for the simultaneous detection of *A*. *marginale* and *T*. *annulata* following Ganguly et al. [14]. Oligonucleotide primers TAF 5′ TGAGTTAACTGTCGCGGATG 3′ and TAR 5′-TGGGCAGGGTGAAGATTAAG 3′ were used to amplify 156 bp fragment of *Tams-1* gene of *T*. *annulata*. While AMF 5′ ACAGGCGAAGAAGCAGACAT 3′ and AMR 5′ ATAAATGGGAACACGGTGGA 3′ were used to specifically amplify 382 base pairs of the *msp5* gene of *A*. *marginale* in positive blood samples. The reaction mixture was prepared in a final volume of 25 μl containing 13 mM Tris-HCl (pH 8.3), 65 mM KCl, 2 mM MgCl_2_, 300 μM of each dNTP, 1 U of AmpliTaq DNA polymerase (Wizbio, Mexico), 0.5 μM of each primer, and 2 μl (50-150ng) of template DNA. Reaction conditions included an initial denaturation step of 94°C for 5 min followed by 30 cycles of denaturation at 94°C for 45 s, primer annealing at 53°C for 50 s and extension at 72°C for 50 s. A final extension at 72°C for 7 min was performed [14]. *T*. *annulata* and *A*. *marginale* positive and negative samples were also used during each PCR reaction as positive and negative controls, respectively.

### DNA sequencing and phylogenetic analysis

Three representative PCR products of each *A*. *marginale* and *T*. *annulata* were randomly selected, purified and sequenced in both directions, using the same primers as for the PCR amplifications. The reaction was performed by a commercial lab (First base Sequencing Service, Selangor, Malaysia). The obtained nucleotide sequences were viewed on FinchTV viewer (Geospiza, Seattle, WA, USA) and checked individually for the sequencing errors. NCBI BLAST algorithm was used to look for the identical and reference sequences. Moreover, the DNA sequences from partial *msp5* gene (346bp) of *Anaplasma marginale* and partial merozoite-piroplasm surface antigen *tams1* gene (118bp) of *Theileria annulata* were deposited in the GenBank database with the following accession numbers OQ571894-96 (*Anaplasma marginale)* and OQ469840-42 (*Theileria annulata*). Multiple sequence alignment was done for the both genes along with the reference sequences and other homological sequences available in the GenBank. The phylogenetic assessment of the current study sequences was done through MEGA-X program utilizing maximum likelihood tree based on kimura-2 parameter model [15].

### Statistical analysis

Significance level was set at P ≤ 0.05. Results were statistically analyzed by using Statistical package Minitab (Minitab, USA). Comparison of *T*. *annulata* or *A*. *marginale* prevalence between various cattle breeds and sampling sites was made by using one way analysis of variance (ANOVA). Epidemiological data were correlated with the presence of each pathogen by the Fischer’s exact test. Whereas the prevalence rates of *T*. *annulata* and *A*. *marginale* was analyzed in cattle samples by using the Chi-square test.

## Results

### Molecular detection of *Theileria annulata* in cattle

During the present study, a total of 298 bovine blood samples were collected from the Bajaur district in Khyber Pakhtunkhwa. Using polymerase chain reaction (PCR), a specific 156 base pair amplicon of the *Tams-1* gene of *Theileria annulata* was detected in 31 of these samples (10.4%). Statistical analysis using the Chi-square test revealed that the prevalence of *Theileria annulata* did not vary significantly among the enrolled cattle (P = 0.08), as presented in Table 1.

**Table 1:** Comparison of *Anaplasma marginal and Theileria ovis* prevalence in in blood samples of cattle collected from Bajaur district of Khyber Pakhtunkhwa. % prevalence of each pathogen is given in parenthesis. P–value in each column indicates the results of Chi-square test calculated for a particular pathogen while p value in row is for the overall study.

	*Anaplasma marginale* positive samples	*Anaplasma marginale* negative samples	*Theileria annulata* positive samples	*Theileria annulata* negative samples	Co infection	Chi square value	P value
	16/298 (5.4%)	282/298 (94.6%)	31/298 (10.4%)	267/298 (89.6%	3/298 (1%)	24.956	P < 0.001***
Chi square value	1.003		0.023				
P value	P < 0.0001 ***		0.8				

P > 0.05 = Non significant; P < 0.001(***) = Highly significant

One way ANOVA results indicated that prevalence of *T*. *annulata* varied significantly between the sampling sites (P = 0.05). Highest parasite prevalence was observed in cattle enrolled from Chawarkand (25%) followed by cattle from Nawagai (20%), Khar (9.6%), Salarzi (8.3%), Mamund (6.8%), Arang (6.7%) and Utman Khel (5.6%). While *T*. *annulata* was not detected in cattle blood samples collected from Charmang areas of Bajaur district in Khyber Pakhtunkhwa (Table 2). However, when the prevalence of *T*. *annulata* was compared between the enrolled cattle breeds, one way ANOVA results indicated that parasite prevalence was not restricted to a particular breed (P = 0.8) (S1 Table).

**Table 2:** Comparison of *Theileria annulata* and *Anaplasma marginale* prevalence in blood samples of cattle collected from various locations in Bajaur district of Khyber Pakhtunkhwa. N represents the total number of cattle samples collected during present study % Prevalence of each pathogen is given in parenthesis. P-value represents the results of one-way ANOVA test calculated for studied parameter.

Cattle sample	N	*Theileria annulata +* ve samples	*Theileria annulata—*ve samples	P-value	*Anaplasma marginale +* ve samples	*Anaplasma marginale*—ve samples	P-value
Khar	73	7/73(9.6%)	66/73(90.4%)	0.05*	3/73(4.1%)	70/73(95.9%)	
Nawagai	30	6/30(20%)	24/30(80%)		1/30(3.3%)	29/30(96.7%)	
Salaezi	36	3/36(8.3%)	33/36(91.7%)		3/36(8.3%)	33/36(91.7%)	
Utman Khel	36	2/36(5.6%)	34/36(94.4%)		1/36(2.8%)	35/36(97.2%)	
Chamarkand	28	7/28(25%)	21/28(75%)		3/28(10.7%)	25/28(89.3%)	0.4
Mamund	73	5/73(6.8%)	68/73(93.2%)		2/73(2.7%)	71/73(97.3%)	
Arang	15	1/15(6.7%)	14/15(93.3%)		3/15(20%)	12/15(80%)	
Charmang	7	0/7(0%)	7(100%)		0/7(0%)	7(100%)	
**Total**	**298**	**31(10.4%)**	**267(89.6%)**		**16(5.4%)**	**282(94.6%)**	

P > 0.05 = Non significant; P < 0.05 = Least significant (*)

### Molecular detection of *Anaplasma marginale* in cattle

During the present study, 298 bovine blood samples were collected from the Bajaur district in Khyber Pakhtunkhwa, and polymerase chain reaction (PCR) was used to detect the presence of a 382 base pair fragment specific to the msp5 gene of A. marginale. The results showed that 16 out of the 298 samples (5.4%) were positive for *A*. *marginale*. Statistical analysis using the Chi-square test revealed a significant variation in the prevalence of *A*. *marginale* among the enrolled cattle (P < 0.001), as presented in Table 1. Further analysis using one-way analysis of variance (ANOVA) demonstrated that *A*. *marginale* infection was not restricted to a specific sampling site (Table 2) or cattle breed (S1 Table), with P values greater than 0.05 for both parameters.

### Co infection of *T*. *annulata* and *A*. *marginale* in sheep and goats

Three cattle (1%) were found to be co-infected with both *T*. *annulata* and *A*. *marginale* during present study. When the overall prevalence of both pathogens was compared between enrolled cattle, Chi square test rest revealed that animals were more susceptible to *T*. *annulata* infection than *A*. *marginale* infection (P = 0.001) (Table 1).

### Molecular characterization and phylogenetic analysis

**Fig 2:**
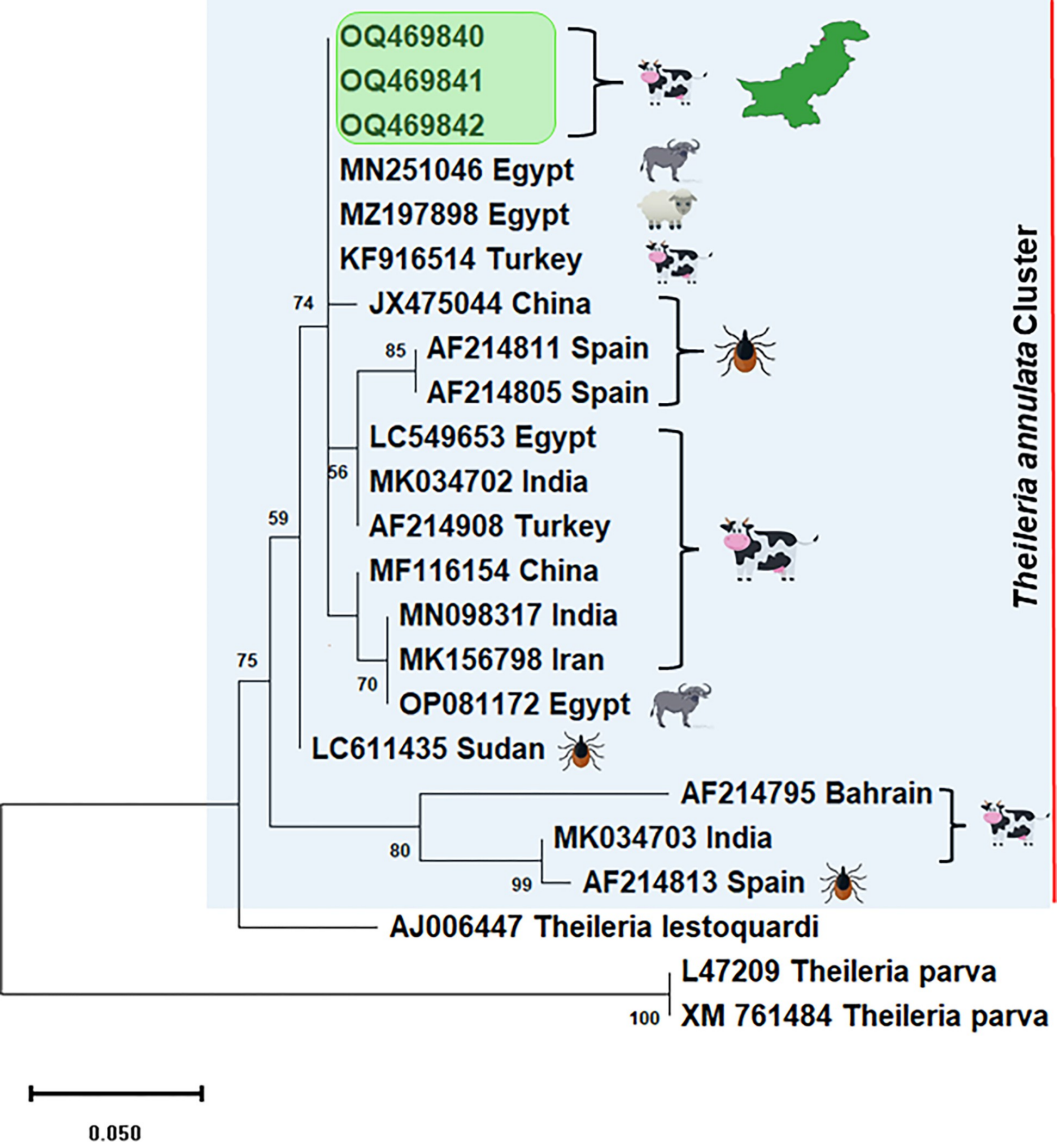
MEGA-X program utilizing maximum likelihood tree based on kimura-2 parameter model was used for the multiple alignments of partial *Tams-1* sequences from *Theileria annulata* isolated in this study and those available in GenBank from other countries around the world. *Theileria lestoquardi* (AJ006447) and *Theileria parva* (L47209 and XM761484) *Tams-1* gene were used as an out group. The three new sequences of *Anaplasma marginale* obtained are highlighted in green box. Scale bar represents 0.50 substitutions per nucleotide position. Bootstrap value is shown as number on each node.

DNA sequencing of three randomly selected positive cattle samples confirmed them to pe part of *Tams-1* gene of *T*. *annulata*. Partial gene sequences were deposited to GenBank under accession numbers OQ469840, OQ469841 and OQ469842. Alignment of partial sequences of *Tams-1* gene from Pakistani cattle revealed a single genotype as all the three sequences generated during present study showed 100% genetic similarity with one another indicating that this *Tams-1* sequence is highly conserved among enrolled cattle (S1 Fig). BLAST analysis revealed 99–100% sequence homology of these sequences with previously deposited *Tams-1* sequences in GenBank. Phylogenetic analysis revealed that the Pakistani isolates clustered together and were similar to *T*. *annulata* isolates deposited from Egypt (MN251046, MZ197898, LC549653 and OP081172) Turkey (KF916514 and AF214908), China (JX475044 and MF116154), Spain (AF214805, AF214811 and AF214813) India (MK034702, MN098317 and MK034703), Iran (MK156798), Sudan (LC611435) and Bahrin (AF214795) (Fig 2).

**Fig 3:**
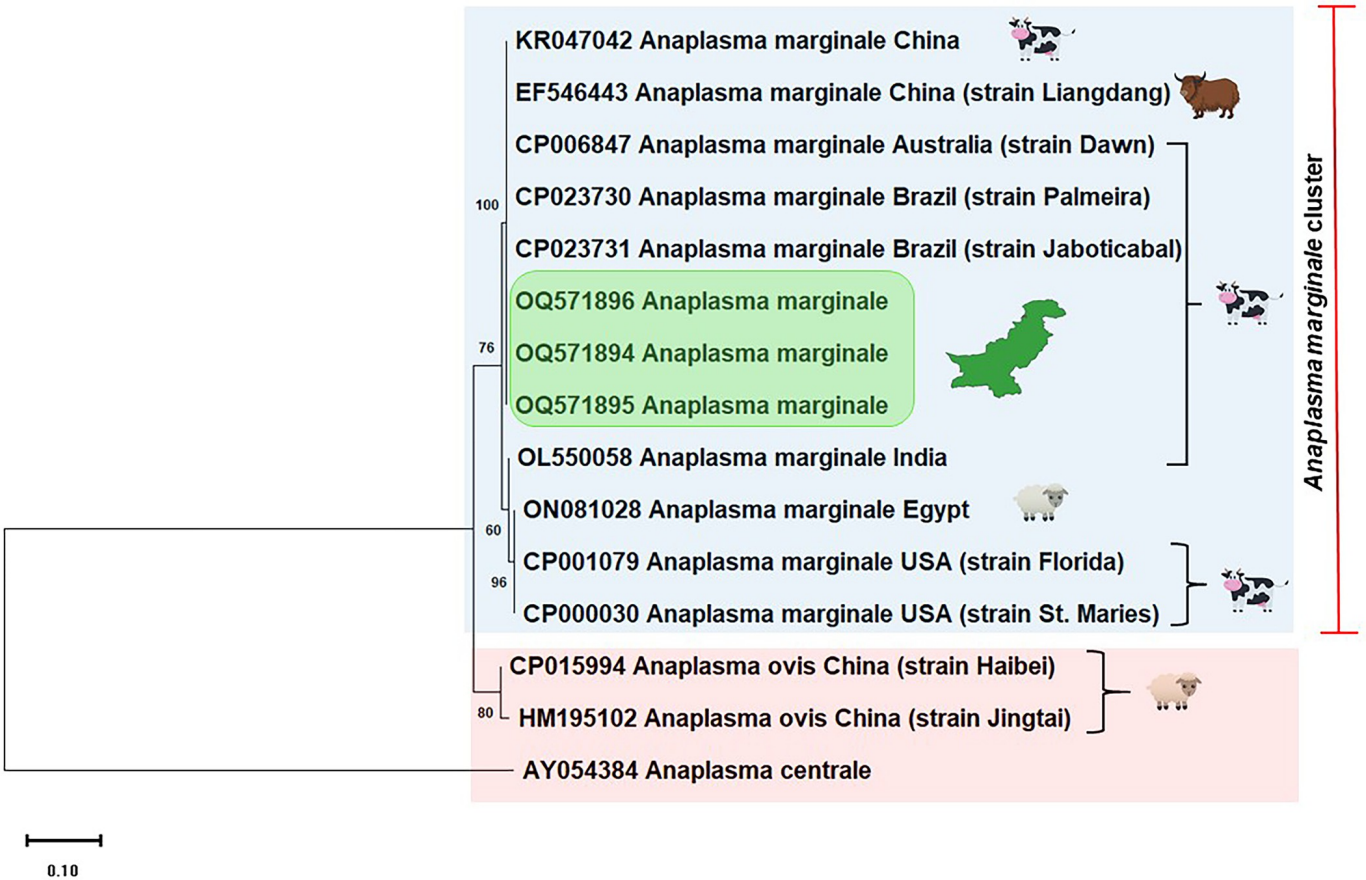
MEGA-X program utilizing maximum likelihood tree based on kimura-2 parameter model was used for the multiple alignments of partial *msp5* sequences from *Anaplasma marginale* isolated in this study and those available in GenBank from other countries around the world. *Anaplasma ovis* (CP015994 and HM195102) and *Anaplasma centrale* (AY054384) *msp5* gene were used as an out group. The three new sequences of *Anaplasma marginale* obtained are highlighted in green box. Scale bar represents 0.20 substitutions per nucleotide position. Bootstrap value is shown as number on each node.

The sequencing of partial *msp5* from three randomly selected positive cattle samples confirmed the *Anaplasma marginale* infection and sequences were deposited to GenBank under accession numbers OQ571894, OQ571895 and OQ571896. Alignment of partial sequences of *msp5* gene from Pakistani cattle revealed a single genotype as all the sequences generated during present study were genetically identical indicating that the conserved nature of *msp5* sequence among the enrolled cattle (S2 Fig). BLAST analysis revealed 99–100% sequence homology of our amplified DNA sequences with those found in GenBank. Phylogenetic analysis revealed that the Pakistani isolates clustered together and were closely related to other *A*. *marginale* isolates previously identified from Brazil (CP023730 and CP023731), Australia (CP006847), China (KR047042 and EF546443), India (OL550058), Egypt (ON081028) and USA (CP001079 and CP000030) (Fig 3).

### Risk factor analysis

Epidemiological data analysis revealed that none of the studied risk factors was found associated either with the prevalence of *T*. *annulata* or *A*. *marginale* among enrolled cattle (P > 0.05) (Table 3).

**Table 3:** Association of *Theileria annulata* and *Anaplasma marginale* prevalence with the studied epidemiological parameters describing cattle characters enrolled during the present study from Bajaur District in Khyber Pakhtunkhwa. N represents the total number of cattle samples collected. % Prevalence of each pathogen is given in parenthesis. P-value represents the results of Fischer Exact test calculated for studied parameter.

Parameters		*Theileria annulata* +ve samples	*Theileria annulata* -ve samples	P-value	*Anaplasma marginale +* ve samples	*Anaplasma marginale*—ve samples	P-value
Age	> 5 years	18/168(10.7%)	150/168(89.2%)	1	8/168(4.8%)	160/168(95.2%)	0.4
	< 5 years	13/130(10%)	117/130(90%)		8/130(6.2%)	122/130(93.9%)	
Sex	Male	6/44(13.6%)	38/44(86.4%)	0.4	1/44(2.3%)	43/44(97.7%)	0.6
	Female	25/254(9.8%)	229/254(90.2%)		15/254(5.9%)	239/254(94.1%)	
Grazing	Present	6/66(9.1%)	60/66(90.9%)	0.8	1/66(1.5%)	65/66(98.5%)	0.2
	Absent	25/232(10.8%)	207/232(89.2%)		15/232(6.5%)	217/232(93.5%)	
Pre-Acaricide spray	Present	8/119(6.7%)	111/119(93.3%)	0.1	6/119(5.1%)	113/119(94.9%)	1
	Absent	23/179(12.8%)	156/179(87.2%)		10/179(5.6%)	169/179(94.4%)	
Tick on animal	Present	23/206(11.2%)	183/206 (88.8%)	0.7	11/206(5.3%)	195/206 (94.7%)	1
	Absent	8/92(8.7%)	84/92(91.3%)		5/92(5.4%)	87/92(94.6%)	
Herd size	< 5	9/70(12.9%)	61/70(87.1%)	0.5	4/70(5.7%)	66/70(94.3%)	1
	> 5	22/228(9.6%)	206/228(90.4%)		12/228(5.3%)	216/228(94.7%)	

P > 0.05 = Non significant

## Discussion

Geologically, Pakistan is located in the warm climate zones of the world, with a hot and humid climate that is suitable for the tick growth [1, 4]. Additionally, uncontrolled crossbreeding with exotic cattle has made Pakistan endemic for tick-borne diseases [5, 16]. Bajaur is a mountainous district in KPK province of Pakistan the population of whom is directly or indirectly dependent on livestock sector for their earning and survival [12] but the prevalence of TBD_S_ in general and bovine theileriosis and anaplasmosis in specific has not been investigated. Hence the present study was designed to report the prevalence of *T*. *annulata* and *A*. *marginale* in various cattle breed from different locations in Bajaur district by using the PCR approach.

During the present investigation, 10.4% of the enrolled cattle were found infected with *T*. *annulata* (Table 1). A few recent reports have documented the molecular prevalence of *Theileria annulata* in Pakistani cattle. Asif et al. [4] has reported that 11.3% of cattle enrolled from Multan district were infected with *T*. *annulata*. Parveen et al. [17] and Ullah et al. [18] had also reported that 20 and 23.7% cattle enrolled from Layyah district in Punjab and central zone of KPK were *T*. *annulata* infected respectively. PCR based presence of *T*. *annulata* in cattle also been reported from other geographic regions of Pakistan and the prevalence ranged from 19 to 66.1% [19–23]. Prevalence of *Theileria annulata* in cattle has also been reported from different countries around the globe. Prevalence of *Theileria annulata* in cattle has been reported to be 83% in Kazakhstan [24], 39% in Sudan [25], 25.4% in Algeria [26], 23.3% in India [27], 18.2% in Northwest China [28], 16.5% in Egypt [29] and 1.9% in Saudi Arabia [30]. These differences in *Theileria annulata* infection rates are due to variations in tick control programs, habitat suitability for ticks, farm management, husbandry practices and abiotic factors of sampling sites [23]. It has also been an established fact now that resource-poor farming communities are at a greater risk due to the lack of systematic acaricide use and limited access to veterinary health care centers leading to a high mortality and morbidity rates [31].

Despite the fact that tropical theileriosis is endemic in many parts of the world, limited information is available in literature regarding the genetic variability in the major merozoite/piroplasm surface antigen (*Tams1*) [32] as most of the molecular epidemiological studies are based on either 18S rRNA [33] or cytochrome b genes [17, 34], that are highly conserved among different isolates globally. We used the three amplified PCR products from the *Tams1* gene of *T*. *annulata* for the phylogenetic analysis. These sequences clustered together and had similarities with the *Tams1* gene sequence of this pathogen deposited from cattle and buffaloes in Egypt (Accession numbers MN251046, MZ197898, LC549653 and OP081172, unpublished data), large ruminants in Turkey (Accession number AF214908) [35], ticks in China (Accession number JX475044) [36], ticks in Spain (Accession numbers AF214805, AF214811 and AF214813) [35], cattle in India (Accession number s MK034702 and MK034703) [37], cattle in Iran (Accession number MK156798, unpublished data), cattle in Sudan (Accession number LC611435) [38] and cattle in Bahrin (Accession number AF214795) [35] (Fig 2). These results indicated that similar *T*. *annulata* sequences are present in various parts of the world.

It was observed during present study that prevalence of *Theileria annulata* varied with the sampling sites (25% in Chamarkand to 5.6% in Utman Khel) (Table 2). Our results are in agreement with Ullah et al. [18] as they had reported that reported higher *Theileria annulata* prevalence in cattle population enrolled from district Mardan than cattle of Charsadda and Peshawar districts in KPK. Parveen et al. [34] had also found significantly higher *T*. *annulata* prevalence in the blood samples of cattle collected from Dera Ghazi Khan district as compared to Lohdran. In a similar study from KPK, Khattak et al. [20] had also reported a significantly higher prevalence of *T*. *annulata* in cattle from Kohat than in cattle from Peshawar district. Similar observations have been reported by Farooqi et al [39], Shahnawaz et al. [40] and Zeb et al. [23] in their studies that were conducted in different geographical regions of Pakistan. All these studies has concluded that differences in the micro and macro climatic conditions of the different agro ecological zone affect the bionomics of the tick vectors; favoring tick fecundity, distribution, abundance, activity and ultimately tick-borne pathogens transmission dynamics that in turn cause the variability in the prevalence rate of bovine theileriosis [23].

During the present study, prevalence of *T*. *annulata* was compared between the three enrolled cattle breeds: Jersey, Achai and crossbred. Our results indicated that parasite prevalence was not limited to a particular cattle breed (S1 Table). This observation is contradictory to the general belief as the local cattle breeds are reported to be tick and TBDs resistant as compared to the exotic breeds [1, 4, 16]. Contrary to our results, recently Atif et al. [41] has also reported that the highest rate of *T*. *annulata* and *A*. *marginale* mixed infection was in Holstein Friesian cattle while the lowest prevalence was observed in indigenous cattle. Similar to our observation, Salih et al. [42] and Ndungu et al. [43] had reported that different cattle breeds enrolled in their studies, conducted in Sudan and Kenya respectively, were equally susceptible to *Theileria* infection.

It was observed during current investigation that 5.4% cattle blood samples collected from Bajaur district were infected with *A*. *marginale* (Table 1). Recently, Asif et al. [1] has reported that 11% cattle located in Multan District of Punjab province in Pakistan were *A*. *marginale* infected. In a similar study, Zafar et al. [5] has reported 9% and 11% prevalence of *A*, *marginale* in cattle from Lohdran and Dera Ghazi Khan Districts in Punjab respectively. Ashraf et al. [9] and Hussain et al. [44] has reported 8.6% and 6.1% infection rate of *A*. *marginale* in cattle blood samples collected from Layyah and Bahawalpur Districts in Punjab respectively. Prior to this investigation, few studies documenting the prevalence of *A*. *marginale* in cattle, has been reported from KPK province. Farooqi et al. [39] had reported 18.3% bovine samples investigated in three distinct zones of KPK were *A*. *marginale* infected. While Turi et al. [45] recorded a prevalence rate of 41.6% of this bacterium in cattle from Peshawar and Lakki Marwat Districts of KPK. Most of the dairy farms in KPK, especially in the rural areas, are poorly managed and old traditional techniques for animal rearing and management are still in practice that leads to poor hygiene and increases the risk of tick infestation and prevalence of TBDs [39]. Prevalence of *A*. *marginale* infection in cattle has also been reported from a number of countries around the globe and the infection rates ranged between 25–100% among the enrolled animals. Prevalence of *A*. *marginale* was reported to be 100% in cattle from Ecuador [46], 29.1% in Turkey [47], 27% in Brazil [48], 25.4% in Tunisia [49] and 15.6% in Nigeria [50]. The prevalence of *A*. *marginale* in cattle varies across different countries, and this variation can be attributed to a range of factors. The effectiveness and scale of tick-control programs implemented in these regions, as well as differences in climate and geography that affect the suitability of certain areas for tick populations, are among the main contributing factors to this variation [49].

Genes encoding major surface proteins (MSPs) are usually targeted for the phylogenetic analysis of *A*. *marginale*. Six MSPs *(MSP1a*, *MSP1b*, *MSP2*, *MSP3*, *MSP4* and *MSP5*) have been identified on *A*. *marginale* derived from bovine erythrocytes. MSPs are involved in host-pathogen interactions and have evolved more rapidly than other nuclear genes because of selective pressures exerted by the host immune system [51]. MSP5 is a highly conserved protein does not vary antigenically during the multiplication of the organism [52]. We used the three amplified PCR products from the *msp5* gene of the rickettsial pathogen for the phylogenetic analysis. The three partial gene sequences generated in this study clustered together and had similarities with the *msp1b* gene sequence of the rickettsial pathogen mainly reported from Brazilian cattle (Accession numbers CP023730 and CP023731, unpublished data), cattle from Australia (Accession number CP006847) [53], cattle from China (Accession number KR047042 and EF546443, unpublished data), cattle from India (Accession number OL550058) [54], sheep from Egypt (Accession number ON081028, unpublished data) and cattle from USA (Accession numbers CP001079 and CP000030) [55] (Fig 3). The findings of our study have expanded upon existing knowledge regarding *A*. *marginale* in Pakistan, and have emphasized the need for further, more in-depth investigations into the genetic diversity of the parasite in various regions of the country. Such investigations would help to establish correlations between genetic diversity and parasite virulence, ultimately leading to better strategies for the prevention and control of *A*. *marginale* infections in cattle.

We observed during this investigation that prevalence of *A*. *marginale* was not limited to a particular sampling site in Bajaur district of KPK (Table 2). Our results are contractor to those of Zafar et al. [5] as they had reported that cattle from Dera Ghazi Khan District in Punjab were more prone to *A*, *marginale* infection than cattle from Lohdran. Similarly Farooqi et al. [39] had also reported a significant variation in *A*. *marginale* infection rate with reference to sampling sites in KPK. The highest bacterial prevalence was recorded in northern zone followed by central and southern zones of this province and they had attributed the differences in prevalence to the different geo climatic conditions and the farm management techniques used in the three study areas.

During the present study, it was observed that the prevalence of *A*. *marginale* was not limited to a particular cattle breed (S1 Table). This observation is in agreement with Zafar et al. [5] as they found that all the cattle breeds enrolled from Lohdran and Dera Ghazi Khan Districts in Punjab were equally susceptible to *A*. *marginale* infection. Our results are contradictory to Ashraf et al. [9], Khan et al. [56] and Tay et al. [57] as all of them had reported higher *A*. *marginale* prevalence in Holstein Friesian breed than in local cattle breeds. They have suggested that as compared to local breeds, Holstein Friesian breed has long and thick hairs making them a preferred host for ticks to infest. Also, the local cattle breeds are considered more tick resistant than exotic breeds [4]. The differences observed in the bacterial prevalence among these studies are probably due to different number of samples that were examined in these studies as well as due to temporal variations during sample collection.

Risk factor analysis revealed that none of the studied parameters in this study was associated with the prevalence of *T*. *annulata* or *A*. *marginale* in cattle enrolled from Bajaur district during present study (Table 3). Our results are contradictory to Atif et al. [41] as they had reported that age, breed, tick infestation, history of tick-borne diseases, frequency of acaricidial application and season were significantly associated with the prevalence of *T*. *annulata* and *A*. *marginale* in cattle from Punjab (Pakistan). Similarly, Selim et al. [29] had reported that risk of theileriosis was elevated in older cattle having tick infestation especially in summers. Parveen et al. [34] had also reported that female cattle, animals housed in close compounds, animals with a tick burden and farms where only cattle were kept were more susceptible to *T*. *annulata* infection. Zafar et al. [5] had also reported a higher prevalence of *A*. *marginale* in cattle living indoors or with other dairy animals in Dera Ghazi Khan District. However, no such relationship was observed in the cattle from Lohdran district in Punjab (Pakistan). Asif et al. [1] had reported that the presence of dogs at dairy farms and dogs having tick burden were the risk factors that were significantly associated with the bovine anaplasmosis in Multan District. A number of factors are responsible for these variations in risk factors associated with the tick borne diseases including the variable number of enrolled animals, sampling season and vector management programs at study sites [9].

In conclusion, our study has revealed that both *T*. *annulata* and *A*. *marginale* infections are endemic in the study site, and that multiplex PCR is an effective method for detecting concurrent field infections. The prevalence of *T*. *annulata* was found to be higher than that of *A*. *marginale* in cattle from the Bajaur district in KPK, and the prevalence of both pathogens was not limited to a particular cattle breed. Furthermore, we observed significant variation in the prevalence of *T*. *annulata* infection between the blood sample collection sites. We recommend that similar large-scale studies be conducted in all regions of Pakistan that have yet to be explored for the prevalence of TBDs, as this would significantly benefit the livestock industry and the economy of Pakistan.

## Supporting information

S1 Fig Tams-1 sequences alignment from Theileria annulata isolates amplified from Pakistani cattle and the sequences deposited in GenBank from various parts of world. Dashes indicate the conserved nucleotide positions. The positions with substitutions in DNA sequence of *Theileria annulata* are represented by different colored nucleotides.
(JPG)

S2 Fig Msp5 sequences alignment from Anaplasma marginale isolates amplified from Pakistani cattle and the sequences deposited in GenBank from various parts of world. Dashes indicate the conserved nucleotide positions. The positions with substitutions in DNA sequence of various *Anaplasma* spp. are represented by different colored nucleotides.
(JPG)

S1 Table Comparison of Theileria annulata and Anaplasma marginale prevalence in blood samples of various cattle breeds enrolled from Bajaur district of Khyber Pakhtunkhwa. N represents the total number of cattle samples collected during present study. % Prevalence of each pathogen is given in parenthesis. P-value represents the results of one-way ANOVA test calculated for studied parameter.
(DOCX)
