# Supplementary figures and images for: Simultaneous molecular detection of Anaplasma marginale and Theileria annulata in cattle blood samples collected from Pakistan-Afghanistan boarder region
Source: PLoS One. 2023 Jul 20;18(7):e0288050. doi: 10.1371/journal.pone.0288050 (PMC10358909; doi:10.1371/journal.pone.0288050)

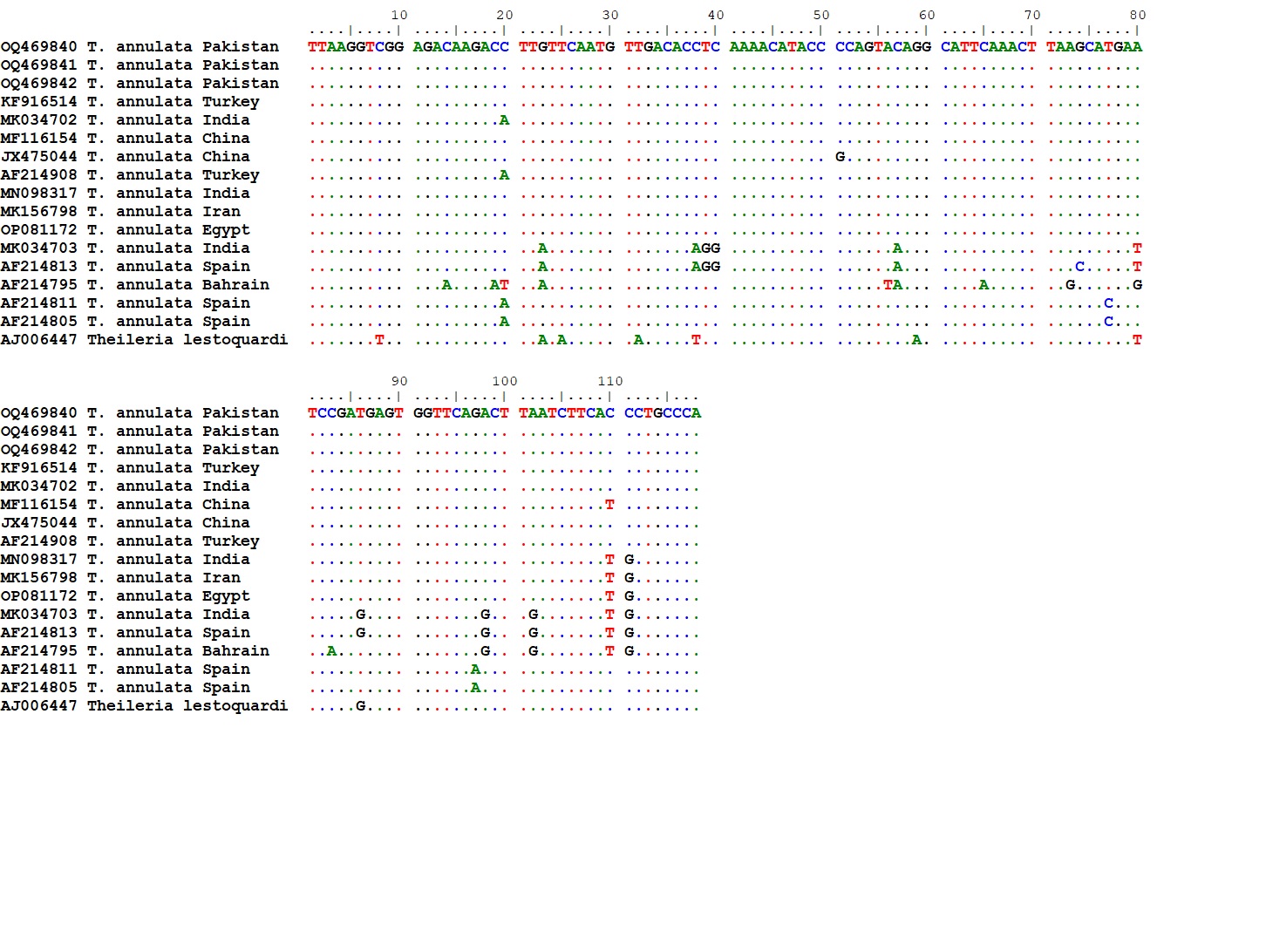

Supplement: S1 Fig — Dashes indicate the conserved nucleotide positions. The positions with substitutions in DNA sequence of Theileria annulata are represented by different colored nucleotides. (JPG) [file pone.0288050.s001.jpg]

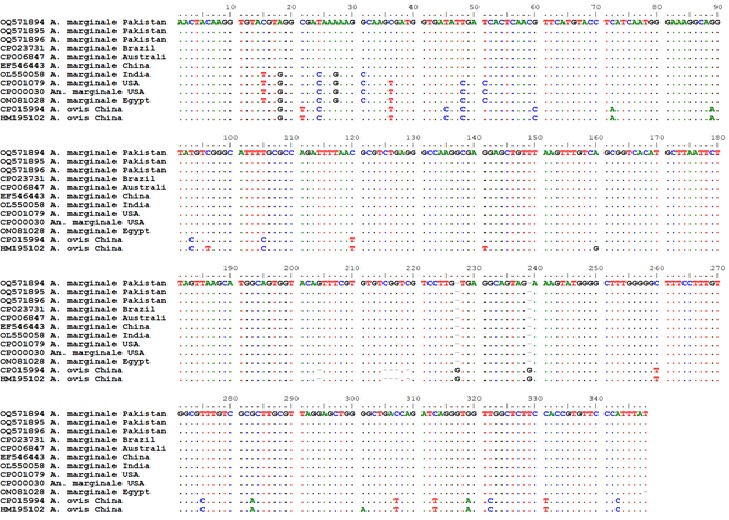

Supplement: S2 Fig — Dashes indicate the conserved nucleotide positions. The positions with substitutions in DNA sequence of various Anaplasma spp. are represented by different colored nucleotides. (JPG) [file pone.0288050.s002.jpg]
