# Supplementary material for: Simultaneous molecular detection of Anaplasma marginale and Theileria annulata in cattle blood samples collected from Pakistan-Afghanistan boarder region
Source: PLoS One. 2023 Jul 20;18(7):e0288050. doi: 10.1371/journal.pone.0288050 (PMC10358909; doi:10.1371/journal.pone.0288050)
Supplement: S1 Table — N represents the total number of cattle samples collected during present study. % Prevalence of each pathogen is given in parenthesis. P-value represents the results of one-way ANOVA test calculated for studied parameter. (DOCX) [file pone.0288050.s003.docx]

**Supplementary Table 1.** Comparison of *Theileria annulata* and *Anaplasma marginale* prevalence in blood samples of various cattle breeds enrolled from Bajaur district of Khyber Pakhtunkhwa. N represents the total number of cattle samples collected during present study. % Prevalence of each pathogen is given in parenthesis. P-value represents the results of one-way ANOVA test calculated for studied parameter.

| **Cattle Breed** | **N** | ***Theileria annulata***  ***+*ve samples** | ***Theileria annulata***  ***-*ve samples** | **P-value** | ***Anaplasma marginale* *+* ve samples** | ***Anaplasma marginale* *-* ve samples** | | | **P-value** | | |
| --- | --- | --- | --- | --- | --- | --- | --- | --- | --- | --- | --- |
| Crossbred | 55 | 5/55(9.1%) | 50/55(90.9%) | 0.8 | 3/55(5.5%) | | | 52/55(94.5%) | |  | |
| Jersey | 88 | 8/88(9.1%) | 80/88(90.9%) |  | 5/88(5.7%) | | 83/88(94.3%) | | | | 1 |
| Achai | 155 | 18/155(11.6%) | 137/155(88.4%) |  | 8/155(5.2%) | | 147/155(94.8%) | | | |  |
| **Total** | **298** | **31(10.4%)** | **267(89.6%)** |  | **16(5.4%)** | | **282(94.6%)** | | | |  |

P > 0.05 = Non significant
